# Cognitive Processing in Non-Communicative Patients: What Can Event-Related Potentials Tell Us?

**DOI:** 10.3389/fnhum.2016.00569

**Published:** 2016-11-14

**Authors:** Zulay R. Lugo, Lucia R. Quitadamo, Luigi Bianchi, Fréderic Pellas, Sandra Veser, Damien Lesenfants, Ruben G. L. Real, Cornelia Herbert, Christoph Guger, Boris Kotchoubey, Donatella Mattia, Andrea Kübler, Steven Laureys, Quentin Noirhomme

**Affiliations:** ^1^Coma Science Group, University and University Hospital of Liège, GIGALiège, Belgium; ^2^Institute of Psychology, University of WürzburgWürzburg, Germany; ^3^French Association of Locked-in Syndrome (ALIS)Paris, France; ^4^Neuroelectrical Imaging and BCI Laboratory, Fondazione Santa Lucia, IRCCSRome, Italy; ^5^School of Life and Health Sciences, Aston Brain Centre, Aston UniversityBirmingham, UK; ^6^Department of Civil Engineering and Computer Science, University of Rome Tor VergataRome, Italy; ^7^Coma Arousal Unit - PMR Department, Nîmes University HospitalNîmes, France; ^8^Institute for Medical Psychology and Behavioural Neurobiology, University of TübingenTübingen, Germany; ^9^Department of Psychiatry, University of TübingenTübingen, Germany; ^10^Department of Biomedical Resonance, University of TübingenTübingen, Germany; ^11^G.Tec Medical Engineering GmbH/Guger Technologies OGGraz, Austria; ^12^Department of Cognitive Neuroscience, Maastricht UniversityMaastricht, Netherlands; ^13^Brain Innovation B.V.Maastricht, Netherlands

**Keywords:** P300, event-related potentials, locked-in syndrome, vegetative state, unresponsive wakefulness syndrome, minimally conscious state

## Abstract

Event-related potentials (ERP) have been proposed to improve the differential diagnosis of non-responsive patients. We investigated the potential of the P300 as a reliable marker of conscious processing in patients with locked-in syndrome (LIS). Eleven chronic LIS patients and 10 healthy subjects (HS) listened to a complex-tone auditory oddball paradigm, first in a passive condition (listen to the sounds) and then in an active condition (counting the deviant tones). Seven out of nine HS displayed a P300 waveform in the passive condition and all in the active condition. HS showed statistically significant changes in peak and area amplitude between conditions. Three out of seven LIS patients showed the P3 waveform in the passive condition and five of seven in the active condition. No changes in peak amplitude and only a significant difference at one electrode in area amplitude were observed in this group between conditions. We conclude that, in spite of keeping full consciousness and intact or nearly intact cortical functions, compared to HS, LIS patients present less reliable results when testing with ERP, specifically in the passive condition. We thus strongly recommend applying ERP paradigms in an active condition when evaluating consciousness in non-responsive patients.

## Introduction

The diagnosis of the presence of consciousness in non-responsive patients due to severe brain injury is a challenging task. Clinical differentiation between disorders of consciousness (DOC) like vegetative state/unresponsive wakefulness syndrome (VS/UWS) and minimally conscious state (MCS) has shown a high rate of misdiagnoses (Andrews et al., 1996; Schnakers et al., 2009b). Likewise, it is difficult to differentiate between patients with DOC and those patients who keep intact consciousness but are unable to move or speak due to a brainstem lesion, as seen in patients with classic locked-in syndrome (LIS; Patterson and Grabois, 1986). The lesion in patients with LIS, touching the corticospinal and corticobulbar pathways, leaves the patient completely unable to make any movements (including speech) except for vertical eye movements or blinking. Nevertheless, in some patients even residual eye movement is impossible (Bauer et al., 1979). This is the complete LIS which can be easily mistaken for a VS/UWS as in both conditions patients have eyes open but are behaviorally non-responsive.

Event related potentials (ERPs) components such as the mismatch negativity (MMN) and the P300 waveform have been used as an index for evaluating cognitive functions both in normal and in pathological neurological conditions (Duncan et al., 2009). The MMN (Näätänen et al., 1978) is elicited when subjects are exposed to a repetitive train of identical stimuli with occasional mismatching stimuli (Luck, 2005) independently of patient’s attention or any behavioral task (Näätänen et al., 2007), and is thought to reflect an automatic process that detects a difference between an incoming stimulus and the sensory memory trace of preceding stimuli (Duncan et al., 2009). Regarding the use of this ERP component for the study of DOC, although it can be detected in patients in MCS or UWS the MMN has not proven useful to differentiate both states (Kotchoubey et al., 2005; Fischer et al., 2010) and the most consistent finding has been its utility to predict recovery from coma (Fischer et al., 1999, 2006; Tzovara et al., 2012).

The P300 ERP component is elicited when subjects detect a different and unpredictable stimulus (target, the oddball) among a train of identical, but irrelevant stimuli (standard) in tasks such as the two-stimulus oddball paradigm (Sutton et al., 1965). The P300 peaks about 250–400 ms post stimulus onset and is more prominent over parietal areas (Polich et al., 1997). Two different components have been described for this waveform: the P3a, which is an earlier frontal component elicited by novel stimuli, and the P3b—on which we will focus in this study—elicited by deviant or infrequent stimuli during the single or two-stimulus oddball paradigm. According to the context-updating theory, the P300-ERP would be an indicator of brain activity which occurs during the revision of mental representations induced by incoming stimuli (Polich, 2007).

The P300 could be a valid and reliable ERP to differentiate between states of consciousness because the amplitude changes when a subject is instructed to focus attention on the deviant stimulus (active condition) as compared to a “just listen” condition (Polich, 2007). Nevertheless, the use of the P300 to distinguish between VS/UWS and MCS has shown contradictory results: some studies have found some differences in this ERP between both groups (Schnakers et al., 2008b; Cavinato et al., 2011; Risetti et al., 2013), while others did not (Kotchoubey et al., 2005; Perrin et al., 2006; Fischer et al., 2010; Real et al., 2016). Covert response to commands in VS/UWS patients has been reported with this ERP component (Chennu et al., 2013). Regarding the assessment of the presence of the P300 waveform in patients with LIS, studies have shown similar responses to healthy controls (HC) both in passive (Perrin et al., 2006) and active (Schnakers et al., 2009a) conditions to semantic paradigms but not in following commands in brain computer interface (BCI) tasks (Lulé et al., 2013).

A recent study has shown a higher prevalence of the auditory P300 evoked by simple tones in an oddball paradigm in healthy subjects (HS) as compared to patients in VS and MCS, but this ERP component showed a low sensitivity to differentiate between VS/UWS and MCS patients (Real et al., 2016). Given the characteristic of LIS patients of presenting with a sub-cortical lesion keeping full consciousness and intact or nearly intact cognitive abilities, it is possible to hypothesize that such a test would be useful for detecting quickly and efficiently the presence of consciousness in these patients. The availability of a reliable and rapid ERP test to differentiate patients with DOC and patients with LIS is an important matter. A previous study has shown that, on average, about two and a half months pass between onset of the brain lesion and diagnosis (León-Carrión et al., 2002). A test, to be used in the context of an acute medical setting, should be sufficiently sensitive to detect signals of conscious mental processing and at the same time short enough to be used despite the fluctuations of vigilance and short attentional span of these patients.

To provide such a test, we investigated an auditory oddball paradigm to elicit the P300 component in a passive and an active condition in a group of HC and in a group of LIS patients. We used a complex-tones paradigm as this kind of tones have shown to elicit a higher reactivity than simple tones for eliciting the P300 in brain injured patients (Kotchoubey et al., 2001). We expect a similar response in both groups with a significant increase in the waveform amplitude when shifting from the passive to the active condition. The presence of these changes would demonstrate the applicability of the paradigm to detect consciousness in non-responsive patients.

## Materials and Methods

### Subjects

Ten (HS, mean age 33.07 ± 11.02 years, 8 male) and 11 patients with LIS (mean age 43 ± 9.42 years, 5 male) participated in the study. HS were recruited and tested at the University of Liège. The inclusion criteria were: age above 18 and no history of neurological or psychiatric disease. Patients were selected following the diagnostic criteria of the American Congress Rehabilitation Medicine for LIS defined by: (1) well sustained eye opening; (2) basic cognitive abilities evident at the examination; (3) severe hypophonia or aphonia; (4) clinical evidence of quadriparesis or quadriplegia; and (5) a primary mode of communication through vertical or lateral eye movement or blinking (American Congress of Rehabilitation Medicine, 1995). Other inclusion criteria for the patients were: good vision and/or hearing, any etiology of brain damage and chronic state of the syndrome. The level of neurological impairment was evaluated with the Patterson and Grabois scale (Patterson and Grabois, 1986). This scale was developed to quantify functional motor recovery in patients with LIS and entails five categories: (1) no recovery, for patients who have no motor recovery and are totally dependent in their care; (2) minimum recovery, for those patients with some minimal voluntary motor return but remaining totally dependent in their care; (3) moderate recovery includes patients with significant motor return allowing them independence in some but not all of their daily activities; (4) full recovery, for patients who gained independence in all daily activities but in who persists some minimal neurological deficit; and (5) no neurologic deficit patients with no residual neurological deficit. Table 1 shows the clinical and demographic characteristics of the patients.

**Table 1:** **Clinical and demographic characteristics of the patients**.

Patient	Gender	Age	Time in LIS (years)	Place of living	Etiology	Neuroimaging findings	Communication	Patterson score
**LIS 1**	F	21	4	I	Stroke	Basilar artery occlusion with ischemia of the brainstem and left thalamus	Eye and head movement	2
**LIS 2**	M	33	12	I	Stroke	Brainstem hematoma extended to cerebellar peduncles	Eye movements, alphabet and PC with adapted contactor	2
**LIS 3**	F	46	18	H	Stroke	Pontine, bulbar, left occipital and cerebellar ischemia	Eye and head movements + PC with adapted contactor	2
**LIS 4**	F	46	4	H	Stroke	Ponto-mesencephalic and right posterolateral bleeding	Eye and head movements, alphabet and PC with adapted contactor	2
**LIS 5**	F	48	4	H	Stroke	Pontine hematoma lateralized to the left	Blinking, eye movements, alphabet and PC with adapted contactor	2
**LIS 6**	M	41	18	H	Stroke	Brainstem lesion	Eye movements, alphabet and PC with adapted contactor	3	
**LIS 7**	M	46	16	I	Stroke	Basilar artery occlusion with infarction of the upper part of the brainstem and left thalamus	Eye movements, alphabet and PC with adapted contactor	2
**LIS 8**	F	48	9	H	Stroke	Brainstem lesion	Eye movements + PC with adapted contactor and speech synthesizer	3
**LIS 9**	M	45	3	H	Stroke	Incomplete ischemic lesion of the brainstem	Eye movements, alphabet, PC with adapted contactor	3
**LIS 10**	M	48	3	H	Stroke	Ponto-mesencephalic and bi- hemispheric infarction.	Eye movements + voice	3
**LIS 11**	M	57	3	H	Stroke	Pontine, left bulbar and right cerebellar ischemic stroke + brainstem edema	Eye movements, alphabet	2

*Abbreviations: H, Home; I, Institution; PC, Personal Computer*.

All LIS patients (except patient 4) were members of the French Association for the locked-in syndrome (ALIS) and were tested in France at their homes or at the Institutions where they lived. Patient 4 was tested at the Neurology Department of the University Hospital of Liège (Liège, Belgium). Informed consent was obtained from all participants or their legal representatives when needed. The study was approved by the Ethics Committee of the University of Liège.

### Experimental Procedure

#### Event Related Paradigm Design

A two-tone auditory oddball paradigm was used to elicit the P3b component. The 420 standard stimuli were a frequent complex tone (standard: 440 + 880 + 1760 Hz) and the 60 deviants a rare complex tone (deviant: 247 + 494 + 988 Hz). All tones had a duration of 50 ms with 5 ms of rise and fall time. The inter-stimulus interval (ISI) was 850 ms. Stimuli were delivered binaurally via in-ear headphones at an intensity of 75 dB, first in a passive condition in which the participants were instructed to just listen to the sounds and second in an active condition, where they were instructed to count the deviants in the stream of standard tones. Each run had a duration of 7.5 min with a 5-min break interval between runs. Thus, the total duration of the experimental procedure was about 20 min.

#### Event Related Potential Recording

Electroencephalogram (EEG) was recorded at a sampling rate of 512 Hz, bandpass filtered between 0.5 and 100 Hz including a notch filter at 50 Hz, using 27 active electrodes mounted in an elastic cap (g.USBamp, g.tec medical engineering GmbH, Austria) placed at the positions Fp1, Fp2, F7, F3, Fz, F4, F8, FC5, FC1, FC2, FC6, T7, C3, Cz, C4, T8, CP5, CP1, CP2, CP6, P7, P3, Pz, P4, P8, O1, O2 following the extended 10-20 system (Oostenveld and Praamstra, 2001). The reference electrode was placed at the left ear lobe and the ground electrode at the AFz position. Four electrodes were used to register vertical electrooculogram (vEOG) and horizontal electrooculogram (hEOG): two were placed above and below of one eye (vEOG) and the other two on the outer canthi of each eye (hEOG).

#### ERP Analysis

EEG recorded signals were pre-processed and analyzed using the NPXlab2012 software (NPX Lab 2012 rel.: 1.9.8.314; Bianchi et al., 2009). Data were bandpass filtered between 1 Hz and 25 Hz and processed with Independent Component Analysis (ICA). Independent components corresponding to ocular artifacts were removed. Trials showing abnormally high voltages (>70 μV in absolute values) were automatically rejected. Datasets with a percentage of valid trials inferior to 80% were not included in the analyses. Trials were segmented from −250 s to 1000 s and averaged to obtain ERPs, with baseline correction by the mean activity from −250 ms to 0 ms pre-stimulus.

Grand averages of each group for each condition and electrodes were computed to identify the peak latency, which was searched in a time window from 250 ms to 450 ms after stimuli onset. Then, the peak amplitude of the difference signal (target vs. non-target) was searched in the individual averages within the range defined by the corresponding grand average peak latency ±75 ms. Finally, we also calculated the area amplitude which is suggested to be more informative and less sensible to noise than the peak amplitude (Luck, 2005; Clayson et al., 2013). The time window used for evaluating the area amplitude (area under the curve) was the same as for the peak amplitude (grand average peak latency ±75 ms).

### Statistical Analysis

#### Detection of the P300

The P300 was identified visually and by running a Student *t*-test on a sample by sample basis at the midline electrodes (Fz, Cz, Pz). Differences were considered statistically significant at *p* < 0.05 with false discovery rate (FDR) correction for multiple comparisons (Benjamini and Hochberg, 1995).

#### Evaluation of Peak Latency, Peak Amplitude and Area Amplitude

For these analyses in the group of patients only those showing the P300 waveform at least in the active condition (LIS 1, 3, 4, 6 and 10) were included. Data were analyzed using the program Statistica (version 10^1^). Normality of the variable distributions was assessed with the Shapiro-Wilk test. We looked for changes in amplitude and latency between conditions (passive listening vs. active counting) in each group (within-group differences) using the Wilcoxon test and we compared the measurements of latency and amplitude between groups with the Mann-Whitney *U*-test. The level of significance was fixed at *p* < 0.05.

## Results

In the HS group, subject 6 had an insufficient number of valid trials due to artifacts (mainly at the Pz electrode) and was discarded. In the group of LIS patients, the patient number 5 stopped the test due to fatigue and could not be included because of insufficient data. From the remaining 10 patients, after the pre-processing, patients 8, 9 and 11 were excluded because they had an insufficient number of valid trials (<80%) due to movement artifacts (pathological and uncontrollable laughing during the session due to pseudobulbar syndrome in patients 8 and 9, and continuous coughing in patient 11 who was tracheotomized). Finally, nine recordings from HS (4 males mean age 32 ± 10 years) and seven recordings from patients with LIS (5 males, mean age 42 ± 10 years) were analyzed to determine the presence of the P300 waveform. In Table 2 the number of trials for the deviant and the standard stimuli, for each condition (active and passive) and for each group (HC and LIS) are reported.

**Table 2:** **Number of trials for the deviant (D) and the standard (S) stimuli, for each condition and each group**.

Group	Active	Passive
**HC**	D = 402/419	D = 398/415	
	ST = 2957/3082	ST = 2927/3076
**LIS**	D = 290/429	D = 328/425
	ST = 2019/3072	ST = 2342/3076

### P300 ERP Component

Seven out of nine HS (77.7%) showed a P300 waveform in the passive condition of the task with a significant difference between the target/non-target stimuli *p* < 0.05. In the active condition, all HS showed an identifiable P3 component with a significant difference between target and non-target stimuli. In the group of LIS patients, three out of seven patients (42.8%) showed the P3 waveform with significant difference target/non target stimuli in the passive condition and five of seven patients (71.4%) in the active condition. Two patients showed a P300 only in the active condition, three patients in both conditions and two patients did not show a significant difference between target and non-target trials in any condition. Table 3 presents the summary of the presence and absence of the P300 component for the HS and LIS patients.

**Table 3:** **ERP results in both groups**.

Healthy controls	P3 (passive)	P3 (passive)	LIS patients	P3 (passive)	P3 (active)
**HC 1**	+	+	**LIS 1**	−	+
**HC 2**	+	+	**LIS 2**	−	−
**HC 3**	+	+	**LIS 3**	−	+
**HC 4**	−	+	**LIS 4**	+	+
**HC 5**	+	+	**LIS 6**	+	+
**HC 6**	−	+	**LIS 7**	−	−
**HC 7**	+	+	**LIS 10**	+	+
**HC 8**	+	+			
**HC 10**	+	+			

*+, component present; −, component absent*.

**Figure 1:**
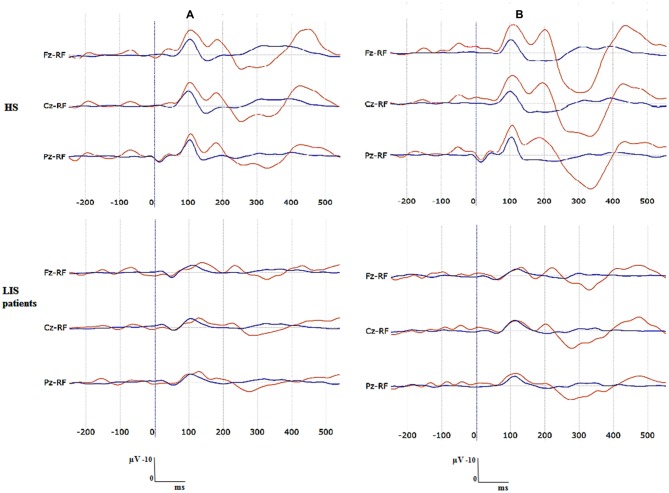
**Grand averaged P300 waveforms for each group at each task condition. (A)** Passive condition, **(B)** active condition. Blue waveform is the average of the standard stimuli. Red waveform is the average of deviant stimuli.

**Figure 2:**
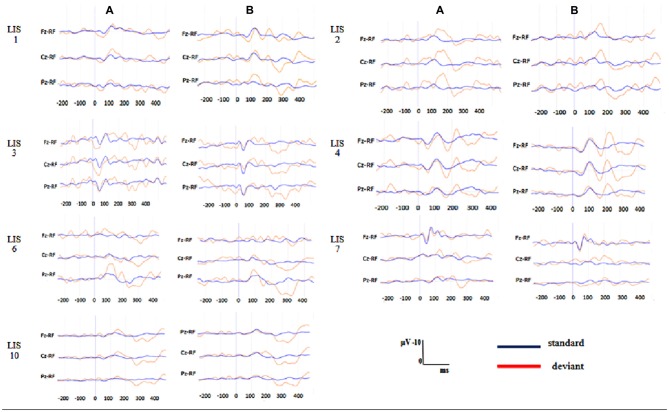
**Individuals P300 waveforms in the patients with locked-in syndrome (LIS) at each task condition. (A)** Passive condition, **(B)** active condition. Blue waveform is the average of the standard stimuli. Red waveform is the average of deviant stimuli.

Figure 1 shows the grand average P300 waveform for both groups at each condition (A = passive condition; B = active condition) and Figure 2 displays the individual waveform for each patient in each task condition.

### Peak Amplitudes, Peak Latencies and Area Amplitudes (Within-Group Differences)

The HS group showed significantly greater peak amplitude and area in the active than in the passive condition (Wilcoxon signed-rank test). This difference was observed for the peak amplitude at the three locations: Fz (*Z* = 2.66, *p* = 0.007), Cz, (*Z* = 2.54, *p* = 0.01), Pz *(Z =* 2.66, *p* = 0.007) and for the area amplitude at Fz (*Z* = 2.66, *p* = 0.007) and Pz (*Z* = 2.54, *p* = 0.01) but not on Cz (*Z* = 1.95, *p* = 0.05). LIS patients did not show significant differences at any location in peak amplitude (Fz: *Z* = 1.21, *p* = 0.22; Cz: *Z* = 1.48, *p* = 0.13; Pz: *Z* = 1.75, *p* = 0.07) and showed only a marginally significant difference at Cz in the area amplitude (Fz: *Z* = 1.78, *p* = 0.07; Cz: *Z* = 2.02, *p* = 0.04; Pz: *Z* = 1.75, *p* = 0.07) measurements between conditions. No significant differences were found in the peak latency between conditions for any group at any location; HS: (Fz: *Z* = 0.86, *p* = 0.38; Cz: *Z* = 0.65, *p* = 0.61; Pz: *Z* = 0.00, *p* = 100); LIS patients: (Fz: *Z* = 0.13, *p* = 0.89; Cz: *Z* = 0.26, *p* = 0.78; Pz: *Z* = 0.13, *p* = 0.89; Table 4).

**Table 4:** **Grand-averages of peak latency, peak amplitude and area amplitude in both groups**.

	Fz		Cz		Pz	
	Passive	Active	Passive	Active	Passive	Active
**Peak latency (ms), mean ± SD**
**HS**	330.8 ± 33.4	316 ± 34.5	329.7 ± 40.1	324.11 ± 41.4	334.4 ± 42.08	334.3 ± 31.4
**LIS**	316.6 ± 18.06	315.6 ± 29.3	317.6 ± 17.5	316.6 ± 26.5	329.8 ± 31	330 ± 26.3
**Peak amplitude (μV) mean ± SD**
**HS**	7.4 ± 4.6	14.7 ± 8.2*	6,26 ± 3.9	12.43 ± 7.4*	5.8 ± 3	11.8 ± 6.05*
**LIS**	5.9 ± 4.4	6.9 ± 5.4	5.7 ± 2.9	7.6 ± 3.4	5.1 ± 1.5	7.9 ± 3.3
Area amplitude (μV*s) mean ± SD
**HS**	0.75 ± 0.45	1.35 ± 0.75*	0.65 ± 0.38	1.11 ± 0.68	0.54 ± 0.26	0.76 ± 0.52*
**LIS**	0.49 ± 0.37	0.66 ± 0.49	0.53 ± 0.40	0.76 ± 0.39*	0.46 ± 0.18	0.70 ± 0.35

*Significant difference at the group between conditions (Wilcoxon test).

### Amplitude and Latency Peaks and Amplitude Area: (Between-Group Differences)

There were no significant differences (Mann-Whitney *U*-test) in any of the three measures (peak amplitude, peak latency and area amplitude) between the HC and the LIS patients neither in the passive nor active condition.

## Discussion

We investigated P300 modulation in a passive and active oddball paradigm in healthy participants and LIS patients to determine whether P300 amplitude increases from passive to active tasks in a similar way in both groups (LIS patients and HC). Contrary to our expectation not all the patients with LIS displayed a P300 in the passive condition—only three out of seven- and the significant increase when passing from passive to active tasks in amplitude and latency could only be confirmed in the group of healthy participants. Nevertheless, more LIS patients had a P300 in the active oddball task as compared to the passive oddball condition.

Thus, the first significant finding was the absence of the P300 waveform in most patients during the passive condition of the task. Although as has been found in previous studies, it is possible that not all HS present this component, even during active tasks (Schorr et al., 2014), with the paradigm used in this study most of the HS showed the response in the passive condition. Factors such as fatigue have shown to be a biological determinant of the P300 component (Polich, 2004). Structural factors linked to the site of injury (ventral pons) might also account for the lack of response in LIS. Norepinephrine inputs from the locus coeruleus located at the posterior region of the pons region have been proposed to account for the P3b component (Nieuwenhuis et al., 2005) which is the component elicited by our paradigm. It is possible that the lesions in LIS patients also affected this area or its projections, producing a decrease of impulses to the temporo-parietal areas and consequently, a decrease in the amplitude or even the disappearance of the P3b.

Medication with blaclofen, an agonist of GABA B receptors widely used for the treatment of spasticity with action on the central nervous system could also contribute to the diminution/disappearance of the P300 component in the LIS patients. It is well known that benzodiazepines, which act on GABA A receptors, can affect the P300 component prolonging latency and decreasing amplitude (Urata et al., 1996). We do not know about studies evaluating the effect of baclofen on the P300 ERP components but, considering its proven central effects, a similar effect could be possible. In our cohort all our patients received baclofen for spasticity (some of them, as patient 2 had a baclofen pump). This hypothesis requires testing, and could be of great importance when evaluating non-responsive patients who receive this drug frequently.

At the active condition all the HS showed the P300 component and a significant increase of the P300 amplitude was also found at the group level. In the group of patients, two of them who did not show the waveform at the passive condition showed it during the active task but, differently from the HS the averaged area amplitude was not significantly higher in the group of patients than in the passive condition. The first explanation of this observation could be related to the small sample size. Nevertheless, it is worth noting that, even though a statistical significant difference could not be detected in the group of LIS patients between conditions or in the comparison with the HS, the mean values of all the measurements were lower in the group of patients.

The aforementioned biological and pharmacological factors might also account for this decreased amplitude of the P300 waveform at the active condition in LIS patients, namely the possible diminution of the locus coeruleus inputs. The P3b generation has been proposed to be mediated by the influence of the locus coeruleus-norepinephrine (LC-NE) system on the allocation of attentional resources and its effects on arousal (Polich, 2007). According to the context updating theory, the decrease of the P300 amplitude would indicate a diminution of the allocation of cognitive resources, mainly working memory and attention to the task. Thus, a direct effect of the alteration of this system would be a decreased capability to engage enough attention required to execute the task and this would be reflected by lower wave amplitude.

Subtle cognitive deficits affecting the performance of the task may also account for our results. In chronic LIS patients previous studies have shown deficits in short and long term memory, in sustained auditory attention (Schnakers et al., 2008a) and in auditory recognition, oral comprehension of complex sentences, delayed visuo-spatial memory, mental calculation and problem solving (Rousseaux et al., 2009). In both studies, the presence of deficits was related to more extended lesions (thalamic or hemispheric). Our sample size does not allow for strong conclusions, but this is a hypothesis which can be tested in future studies by including neuropsychological testing along with ERP measurements.

The order of presentation of the paradigm may also have affected the results. The active condition being always recorded after the passive, some participants may have experienced fatigue, reducing the amplitude of the active ERP and the difference between both conditions.

Finally, auditory impairment, reported in about 20% of patients with LIS in previous studies (Lugo et al., 2015) might also have played a role. In these cases, the possibility of eliciting the P300 by using other sensory modalities such as the vibrotactile stimulation would also be possible and has been already shown to be feasible in LIS patients (Kaufmann et al., 2013; Lugo et al., 2014).

Some limitations of this study need to be mentioned: we lost a substantial amount of data (records from three patients) due to artifacts caused by disease related issues such as uncontrolled coughing or involuntary movement. This is important as these artifacts may restrict the routine application of any ERP paradigm. However, new movement resistant EEG recording equipment may reduce this problem in the future. Further the sample size was low, thus preventing generalization of results. Due to the difficulties in recruiting LIS patients and conducting EEG measurements in a field environment small sample sizes are likely to remain an issue.

In conclusion, in this study we found in a group of LIS patients, though consciously aware, a reduced responsiveness to stimulation with a passive and an active auditory oddball paradigm and only in HS the P300 increased as expected from the passive to the active condition. ERP paradigms need to be further refined and tested in a larger sample of this patient group and also with genuine DOC patients before conclusions about their applicability for diagnosis can be drawn. Alterations have been suggested in the topography, latency and amplitude of ERPs in VS/UWS and MCS patients (Kotchoubey et al., 2005; Real et al., 2016). Our findings, although limited in their generalizability by the small sample size, allows us to suggest the possibility that, in conscious patients with localized damage, these potentials may be completely absent due to multiple factors. These factors include location of the structural lesion, sensory deficits, administered medication, fluctuating vigilance and reduced attention span. In future research ERP paradigms have to be further investigated in patients with LIS and DOC, but also in HS to establish a normative database. The most reliable and discriminative paradigms can then be compiled to a reliable and clinically relevant battery for the assessment of the level of consciousness.

## Author Contributions

QN, ZRL, AK, SV, BK, CH, RGLR: concept and design; ZRL, DL, FP, CG, QN: acquisition of data; ZRL, LRQ, LB, QN: analysis and interpretation of data; ZRL, AK, QN: drafting of the manuscript; AK, DM, QN, BK, SV, RGLR, CH, SL: critical revision of the manuscript for important intellectual content.

## Funding

This research was supported by the French Association of Locked in Syndrome (ALIS), the Belgian National Funds for Scientific Research (FNRS), the European Commission (European ICT Programme Project FP7-247919 DECODER), FEDER structural fund RADIOMED-930549, Fonds Léon Fredericq, the James McDonnell Foundation, the Mind Science Foundation, the French Speaking Community Concerted Research Action (ARC-06/11-340), the University of Würzburg and the University and University Hospital of Liège. The funding sources are not liable for any use that may be made of the information contained therein.

## Conflict of Interest Statement

The authors declare that the research was conducted in the absence of any commercial or financial relationships that could be construed as a potential conflict of interest.

## Footnotes

^1^www.statsoft.fr
